# Simultaneous polychromatic flow cytometric detection of multiple forms of regulated cell death

**DOI:** 10.1007/s10495-019-01528-w

**Published:** 2019-02-20

**Authors:** D. Bergamaschi, A. Vossenkamper, W. Y. J. Lee, P. Wang, E. Bochukova, G. Warnes

**Affiliations:** 1 Centre for Cutaneous Research and Cell Biology, The Blizard Institute, Barts and The London School of Medicine and Dentistry, Queen Mary London University, 4 Newark Street, London, E1 2AT UK; 2 Centre for Immunobiology, The Blizard Institute, Barts and The London School of Medicine and Dentistry, Queen Mary London University, 4 Newark Street, London, E1 2AT UK; 3 Centre for Genomics and Child Health, The Blizard Institute, Barts and The London School of Medicine and Dentistry, Queen Mary London University, 4 Newark Street, London, E1 2AT UK; 4 Flow Cytometry Core Facility, The Blizard Institute, Barts and The London School of Medicine and Dentistry, Queen Mary London University, 4 Newark Street, London, E1 2AT UK

**Keywords:** Regulated cell death, Apoptosis, Necroptosis, RIP1-dependent apoptosis, Autophagy, Hyper-activation of PARP, DNA damage response, Parthanatos, Flow cytometry

## Abstract

Currently the study of Regulated Cell Death (RCD) processes is limited to the use of lysed cell populations for Western blot analysis of each separate RCD process. We have previously shown that intracellular antigen flow cytometric analysis of RIP3, Caspase-3 and cell viability dye allowed the determination of levels of apoptosis (Caspase-3^+ ve^/RIP3^− ve^), necroptosis (RIP3^Hi + ve^/Caspase-3^− ve^) and RIP1-dependent apoptosis (Caspase-3^+ ve^/RIP3^+ ve^) in a single Jurkat cell population. The addition of more intracellular markers allows the determination of the incidence of parthanatos (PARP), DNA Damage Response (DDR, H2AX), H2AX hyper-activation of PARP (H2AX/PARP) autophagy (LC3B) and ER stress (PERK), thus allowing the identification of 124 sub-populations both within live and dead cell populations. Shikonin simultaneously induced Jurkat cell apoptosis and necroptosis the degree of which can be shown flow cytometrically together with the effects of blockade of these forms of cell death by zVAD and necrostatin-1 have on specific RCD populations including necroptosis, early and late apoptosis and RIP1-dependent apoptosis phenotypes in live and dead cells. Necrostatin-1 and zVAD was shown to modulate levels of shikonin induced DDR, hyper-action of PARP and parthanatos in the four forms of RCD processes analysed. LC3B was up-regulated by combined treatment of zVAD with chloroquine which also revealed that DNA damage was reduced in live cells but enhanced in dead cells indicating the role of autophagy in maintaining cell health. This approach to RCD research should be a great advance to understanding the mechanisms of drugs and their effects upon RCD populations.

**Electronic supplementary material:**

The online version of this article (10.1007/s10495-019-01528-w) contains supplementary material, which is available to authorized users.

## Introduction

The evolution of the cell death classification from simple macroscopic morphological characteristics termed Type I–III modes of cell death, including apoptosis, autophagy and necrosis, respectively to now include biochemical and functional parameters has refined and reclassified the many forms of Regulated Cell Death (RCD) [1–4]. These include intrinsic and extrinsic apoptosis (often expressing active caspase-3), immunogenic cell death (ICD) or viral-infection induced translocation of calreticulin (CALR) from the ER to the outer plasma membrane leaflet [5], autophagy (up-regulation of LC3B) and lysosome dependent cell death (caused by cathepsins) [4], necroptosis (up-regulation of RIP3) [6], MPT or mitochondrial “permeability transition pore complex”—driven necrosis (Oxidative stress and calcium overload) [4], parthanatos (hyper-activation of Poly (ADP-ribose) polymerase or PARP) caused by excessive DNA damage (e.g. by H2AX) [7–9], pyroptosis (up-regulation of Caspase-1 and plasma membrane pre-formation by gasdermin) [10], ferroptosis which is ROS and iron dependent lipid peroxidation and mitochondrial dysfunction controlled by GPX4 or GSH-dependent enzyme glutathione peroxidase [11], NETotic cell death (granulocyte H2AX) and entotic cell death or loss of integrin signalling in epithelial cells [4]. This classification currently leaves out RIP1-dependent apoptosis RIP3/Caspase-3 [6, 12–14], the DNA Damage Response (DDR) [15] and ER stress, which causes autophagy and apoptotic cell death [4, 16], with cell senescence [17] and mitotic catastrophe being classed as a non-lethal process [18].

Determination of the type of RCD prevalent in a cell sample is currently determined by Western blot analysis of a lysed cell population and use of the antibody against a specific intracellular target antigen such as Caspase-3, RIP1, RIP3, LC3B, HA2X or PARP to determine which RCD is predominately present and to what degree. The recent [19] development of a three colour flow cytometry assay in this laboratory targeting Caspase-3, RIP3 and cell viability has allowed the detection and quantification of multiple forms of RCD including, necroptosis, early and late apoptosis and RIP1-dependent apoptosis simultaneously in a single cell population.

The addition of PARP and H2AX to identify parthanatos, DDR and H2AX hyper-activation of PARP in live and dead cells allows the detection of 24 distinct populations [19] and demonstrated that double negative (DN) cells for RIP3 and caspase-3 undergo mainly parthanatos [19].

In this study we have added markers for autophagy (LC3B) and ER stress (PERK) making it possible to detect 248 RCD Jurkat cell populations flow cytometrically [4, 20–22]. This multiplexing was particularly useful for investigating the mode of action of drugs. For example the drug shikonin a naturally occurring naphthoquinone which [19, 23–26] is known to induce apoptosis as well as necroptosis in Jurkat cells, so we investigated the modulation of the incidence of these and other types of RCD including RIP1-dependent apoptosis, DDR, hyper-action of PARP and parthanatos by pan-caspase and RIP protein blocker zVAD and necrostatin-1 respectively [26, 27]. Extending the protocol to include analysis of autophagy was investigated how this form of RCD maintains cell health by measurement of the incidence of DNA damage in live and dead autophagic Jurkat cells [28]. Other cell types not included in this study were also analysed giving similar distribution of the markers employed in this study, these included K562 (myeloid leukemic cell), NTERT keratinocytes and hepatocyte cell line Huh7.5-SEC14L2.

## Materials and methods

### Cell cultures

Jurkat cells (human acute T cell leukaemia cell line) were grown in RPMI 1640 medium with 10% FBS (Invitrogen, UK) at 37 °C with 5% CO_2_ either untreated or treated with shikonin (0.5 µM, Santa Cruz, USA) or chloroquine (CQ, 100 µM, Sigma Chemicals, UK) for 24 h. Cells were also pre-treated with pan-caspase blocker zVAD (20 µM, Enzo Life Sciences, USA) and/or necroptosis blocker necrostatin-1 (60 µM Cambridge Bioscience, UK) for 2 h before drug treatment.

### Flow cytometry analysis

Harvested Jurkat cells were labelled with fixable live dead stain, Zombie NIR (Near Infra-Red) (BioLegend, UK) at RT for 15 min. Washed cell pellets were fixed in Solution A (CalTag, UK) for 15 min at RT. Washed cells were then permeabilised in 0.25% Triton X-100 (Sigma, UK) for 15 min at RT. Cells (1 × 10^6^) were subsequently incubated for 20 min at RT with anti-LC3B monoclonal antibody (1:400 dilution, Cat No 3868, Cell Signalling Technology Inc., US). Washed cells were then labelled with 0.125 µg of secondary fluorescent conjugate Alexa Fluor 647 donkey anti-rabbit IgG (Invitrogen, UK). Washed cells (0.5 × 10^6^) were then labelled 2 µl of anti-PERK-AF488, RIP3-PE (clone B-2, Cat. No. sc-374639, Santa Cruz, USA), PARP-PE-CF-595 (Becton Dickinson, UK), H2AX-PE-Cy7 (BioLegend, UK) and anti-active caspase-3-BV650 (Becton Dickinson, UK) for 20 min at RT. Washed cells were resuspended in 400 µl PBS and analysed on a ACEA Bioscience Novocyte 3000 flow cytometer (100,000 events).

### RCD phenotype strategy

**Fig. 1:**
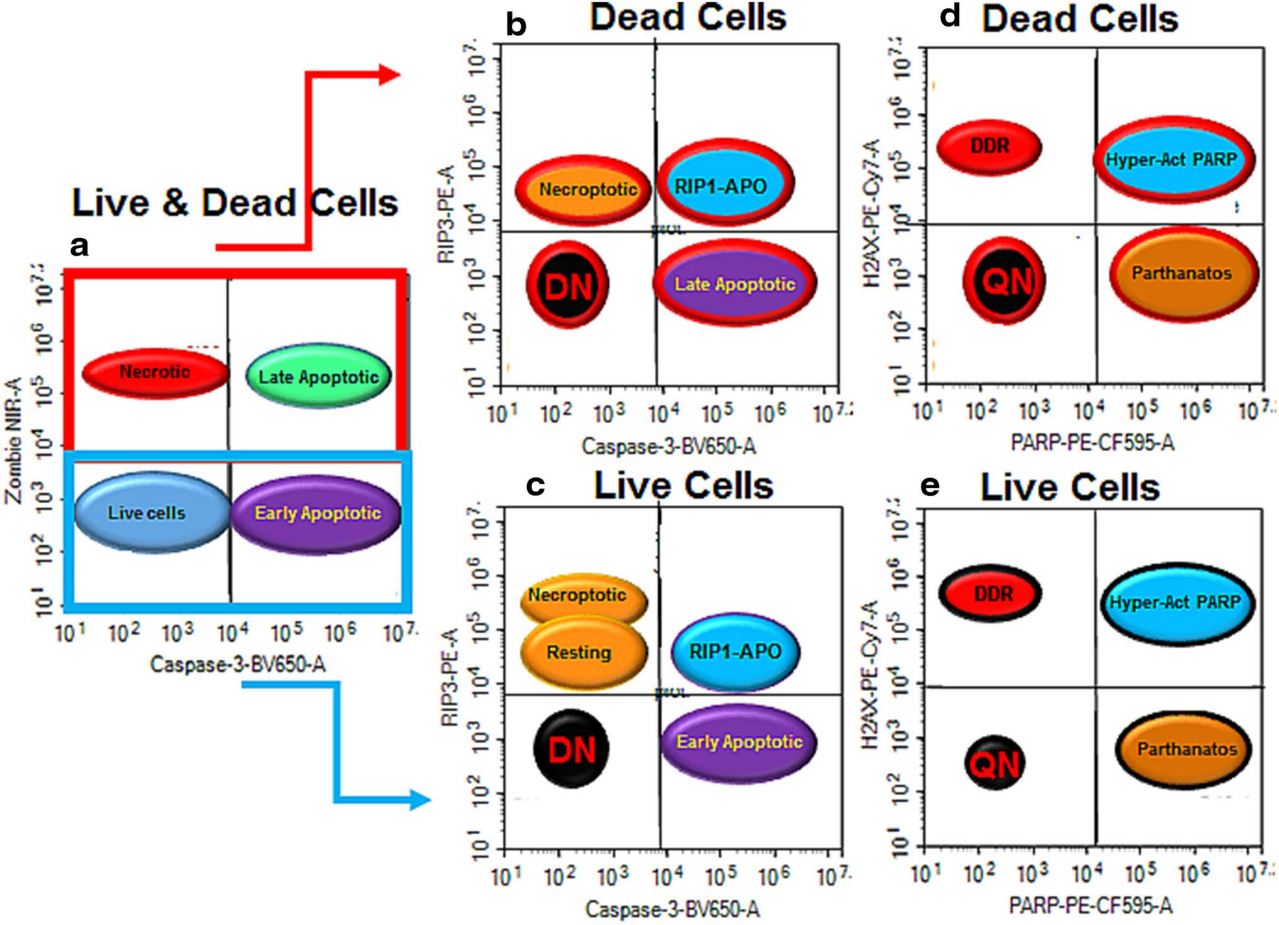
Schematic of immunophenotyping of RCD. Live (including early apoptotic) and dead (including late apoptotic) cells were gated from a Zombie NIR vs. Caspase-3-BV650 dot-plot (**a**). Live and dead necroptotic cells were defined as Caspase-3^− ve^/RIP3^high + ve^, early or late apoptotic (Caspase-3^+ ve^/RIP3^− ve^), RIP1-dependent apoptotic (RIP1-APO, Caspase-3^+ ve^/RIP3^+ ve^) and Double Negative (DN, Caspase-3^− ve^/RIP3^− ve^) (**b, c**). Then each of these live and dead cell populations were then gated on a H2AX vs. PARP dot-plots to show the incidence of DDR (H2AX^+ ve^/PARP^− ve^), hyper-activation of PARP (H2AX^+ ve^/PARP^+ ve^), parthanatos (H2AX^− ve^/PARP^+ ve^) and QN (H2AX^− ve^/PARP^− ve^), (**d, e**)

Cells were gated on FSC vs. SSC removing the small debris near the origin with single cells being gated on a FSC-A vs. FSC-H dot-plot. Cells were then gated on a dot-plot of Caspase-3-BV650 vs. Zombie NIR with a quadrant placed with live cells in the double negative quadrant (lower left), with Caspase-3-BV650^+ ve^/Zombie NIR^− ve^ (lower right) indicating early apoptotic cells, with late apoptotic and necrotic cells gated to include Caspase-3-BV650^+ ve^/Zombie NIR^+ ve^ and Caspase-3-BV650^− ve^/Zombie NIR^+ ve^ (see Fig. 1a). Live (including early apoptotic) and dead cells were gated separately and analysed in RIP3 vs. Caspase-3 dot-plots with RIP3^+ ve^/Caspase-3^− ve^ indicating normal resting cells or necroptosis when RIP3 Median Fluorescence Intensity (MFI) was up-regulated. RIP3^− ve^/Caspase-3^+ ve^ cells indicate those that have undergone apoptosis. Double positive events indicate cells of the RIP1-dependent apoptosis phenotype (Fig. 1b, c). This is an assumed observation, as RIP3 is associated with RIP1 in the formation of the necrosome (RIP1 and RIP3 were observed to be present in PBMNC, data not shown [23]). For each of these phenotypes the incidence of DDR (H2AX^+ ve^/PARP^− ve^), H2AX hyper-activation of PARP (H2AX^+ ve^/PARP^+ ve^), parthanatos (H2AX^− ve^/PARP^+ ve^) and quadruple negative (QN) populations were determined (Fig. 1d, e).

Likewise for autophagy (LC3B^+ ve^) and ER stress (PERK^+ ve^) were phenotyped then LC3B^+ ve^/PERK^− ve^ (live and dead cells) were analysed for RIP3/Caspase-3 to determine the incidence of resting/necroptosis, early or late apoptosis, RIP1-dependent apoptosis and DN phenotypes. Live and dead autophagic cells were also phenotyped for H2AX and PARP to determined levels of DDR, H2AX hyper-activation of PARP and parthanatos (Fig. 6).

### Statistical analysis

All experiments n = 3, with results expressed as mean ± SEM for percentage positive or Median Fluorescent Intensity (MFI). Student *t* tests were performed in GraphPad software Inc., USA with *P* ≥ 0.05 not considered significant (NS), *P* ≤ 0.05*, *P* ≤ 0.01** ,  *P *< 0.001*** when treated cells were compared to untreated.

## Results

### Induction of apoptosis and necroptosis

**Fig. 2:**
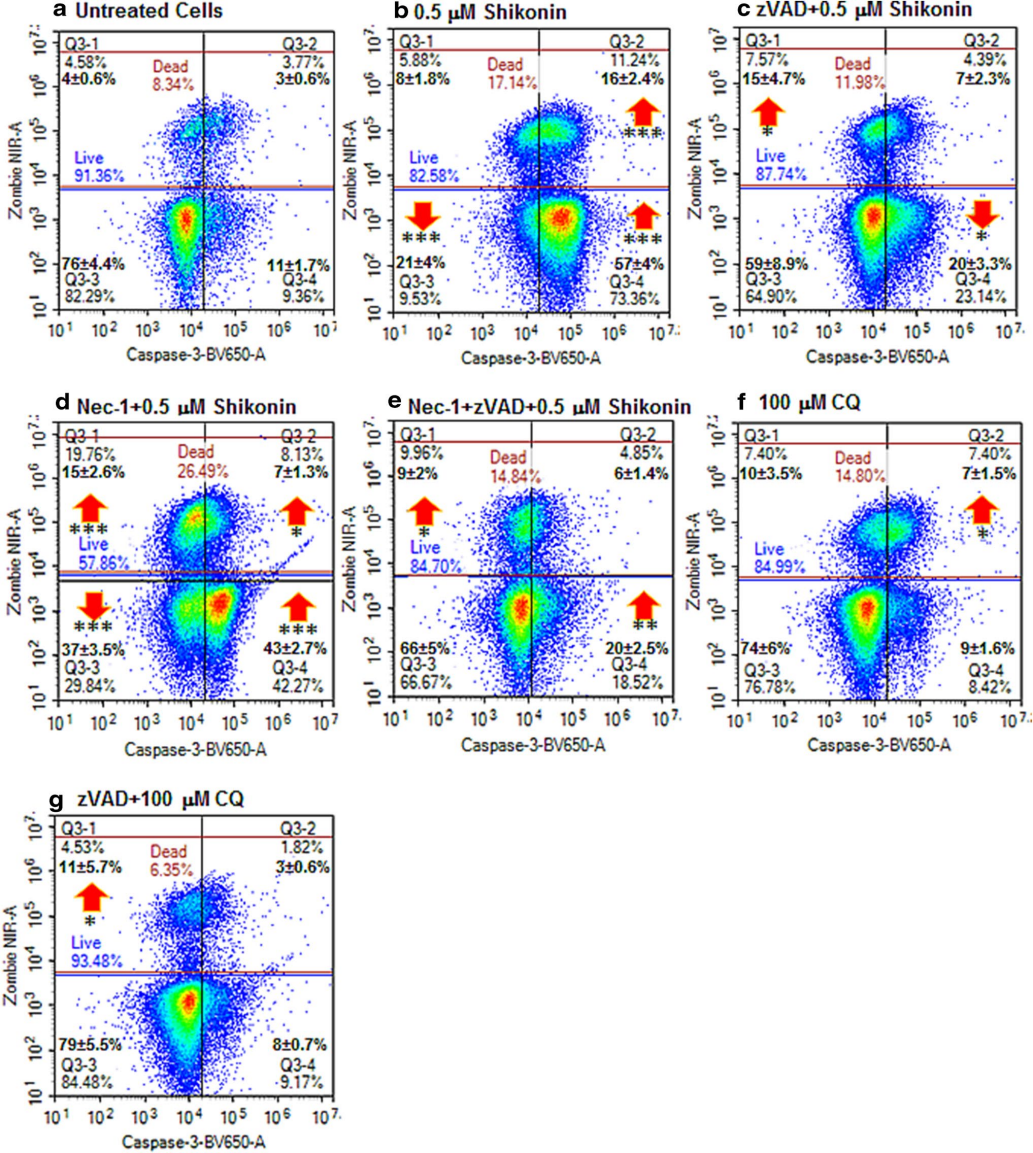
Cell death and caspase-3 activation assay. Jurkat cells were untreated (**a**), treated with 0.5 µM shikonin for 24 h (**b**), pre-treated with 20 µM zVAD for 2 h then with 0.5 µM shikonin for 24 h (**c**), pre-treated with 60 µM necrostatin-1 (Nec-1) for 2 h then with 0.5 µM shikonin for 24 h (**d**), pre-treated with 20 µM zVAD and 60 µM necrostatin-1 (Nec-1) for 2 h then with 0.5 µM shikonin for 24 h (**e**), treated with 100 µM CQ for 24 h (**f**), pre-treated with 20 µM zVAD for 2 h then with 100 µM CQ for 24 h (**g**). n = 3, student *t* test NS (not significant), *P* < 0.05*, *P* < 0.01**, *P* < 0.001***, with arrows indicating change compared to untreated cells

**Fig. 3:**
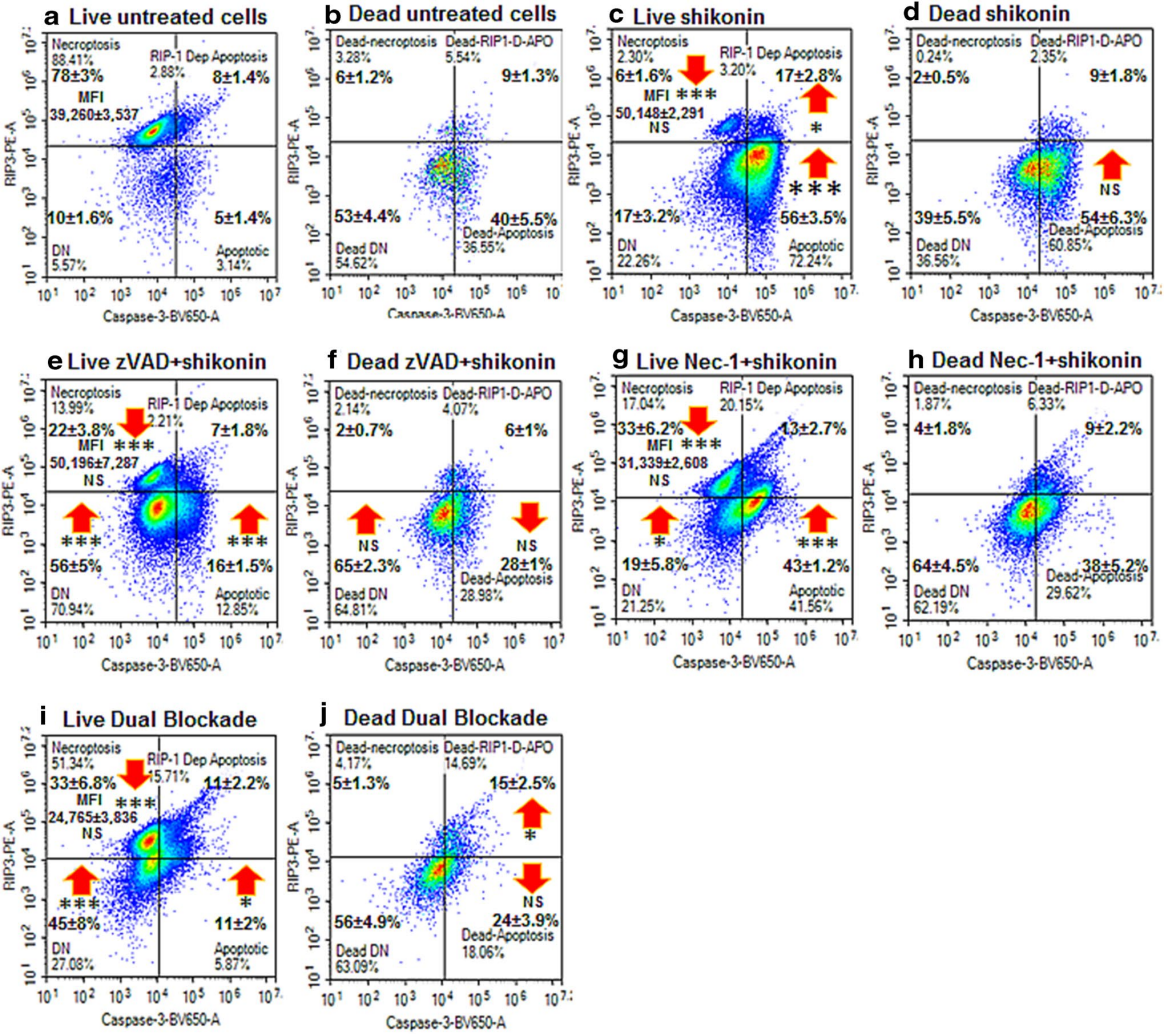
RIP3 and caspase-3 activation assay. After gating on live and dead cells from a Zombie NIR vs. Caspase-3-BV650 dot-plot untreated live (**a**) and dead (**b**) Jurkat cells were analysed on a RIP3-PE vs. Caspase-3-BV650 dot-plot with resting or necroptotic phenotype indicated by RIP3^high + ve^/Caspase-3^− ve^ with the RIP3 Median Fluorescent Intensity (MFI) up-regulated during necroptosis. The apoptosis phenotype (RIP3^− ve^/Caspase-3^+ ve^), RIP1-dependent apoptosis (RIP3^+ ve^/Caspase-3^+ ve^) and double negative (RIP3^− ve^/Caspase-3^− ve^). Live and dead cells treated with 0.5 µM shikonin for 24 h (**c, d**) pre-treated with 20 µM zVAD for 2 h then with 0.5 µM shikonin for 24 h (**e, f**) pre-treated with 60 µM Nec-1 for 2 h then with 0.5 µM shikonin for 24 h (**g, h**) pre-treated with 20 µM zVAD and 60 µM Nec-1 for 2 h then with 0.5 µM shikonin for 24 h (**i, j**) n = 3, student *t* test NS (not significant), *P* < 0.05*, *P* < 0.01**, *P* < 0.001***, with arrows indicating change compared to untreated cells

Shikonin induced a high degree of early (57%, Caspase-3^+ ve^/Zombie NIR^− ve^) and late apoptosis (16%, Caspase-3^+ ve^/Zombie NIR^+ ve^) compared to untreated Jurkat cells (Fig. 2a, b) [24]. Live cells showed necroptosis (RIP3^high + ve^/Caspase-3^− ve^), with an up-regulation of RIP3 (28%, Median Fluorescent Intensity MFI 50,148 ± 2291), early apoptosis (RIP3^− ve^/Caspase-3^+ ve^, 56%) and RIP1-dependent apoptosis (RIP3^+ ve^/Caspase-3^+ ve^, 17%) compared to untreated cells (Fig. 3a, c) [23, 26]. Dead shikonin treated cells like untreated showed only a high degree of late apoptosis (Fig. 3b, d) [23].

**Fig. 4:**
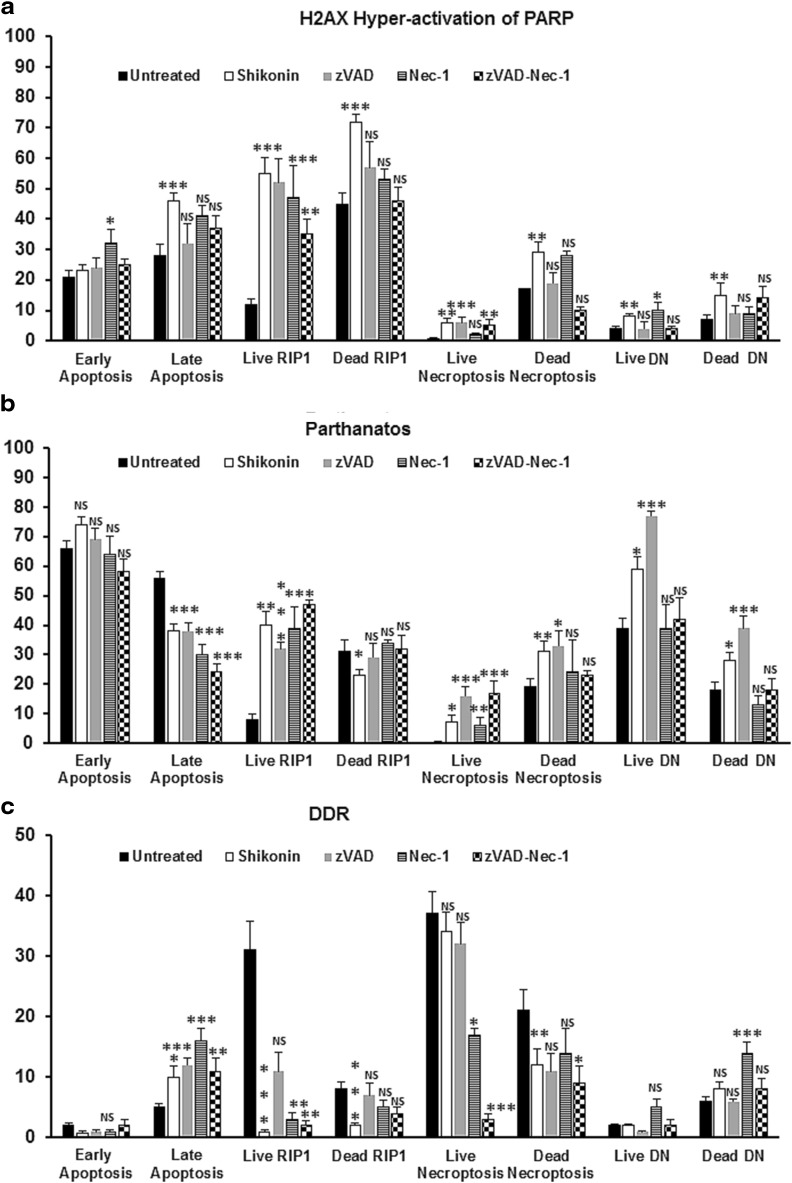
Hyper-activation of PARP, parthanatos and DDR assay. Jurkat untreated, treated with 0.5 µM shikonin or pre-treated zVAD (20 µM) and or Necrostatin-1 (60 µM) for 2 h then incubated with 0.5 µM shikonin for 24 h. After gating on live and dead cells from a Zombie NIR vs. Caspase-3-BV650 dot-plot untreated or treated live and dead Jurkat cells were analysed on a RIP3-PE v Caspase-3-BV650 dot-plot. From which early and late apoptotic, necroptotic/resting, RIP1-dependent apoptotic and double negative (DN) populations were analysed for H2AX and PARP, see Figs. 1S, 2S for detailed information. The incidence of hyper-activation of PARP (**a**), parthanatos (**b**) and DDR (**c**) were determined for all populations listed above. n = 3, student *t* test NS (not significant), *P* < 0.05*, *P* < 0.01**, *P* < 0.001*** compared to untreated cells

All live treated (except early apoptotic) populations showed increased hyper-activation of PARP (DP, H2AX^+ ve^/PARP^+ ve^) and parthanatos (H2AX^− ve^/PARP^+ ve^) with a decreased incidence of the quadruple negative population for all live cell phenotypes compared to untreated cells (QN, Fig. 4a, b, Fig. 1S i–l). While the two live apoptotic populations showed a decrease in DDR (Fig. 4c, Fig. 1Si, j). All dead cell phenotypes also showed an increase hyper-activation of PARP (H2AX^+ ve^/PARP^+ ve^) and a decrease in the incidence of the QN populations compared to untreated dead cells (Fig. 4a, Fig. 1Sm–p). Dead cell parthanatos (unlike live cells) was decreased in both dead apoptosis populations but like live cells this was increased in the necroptotic and DN populations (Fig. 4b, Fig. 1Sm–p). The late apoptotic population however, also showed an increase in DDR, while this was decreased in the necroptotic and RIP1-dependent apoptotic cells compared to untreated dead cells (Fig. 4c, Fig. 1Sm–o).

### zVAD blockade of apoptosis

Pre-treatment of cells with pan-caspase blocker zVAD (20 µM) before shikonin reduced the incidence of early and late apoptosis but increased necrosis (Caspase-3^− ve^/Zombie NIR^+ ve^, Fig. 2b, c) [23]. Blockade of shikonin with zVAD increased the incidence of necroptosis compared to treated cells (Fig. 3e). RIP1-dependent apoptosis was reduced while the DN population increased compared to shikonin treatment (Fig. 3a, c, e). Dead cells also showed a decrease in late apoptosis and an increase in DN population compared to drug treatment (Fig. 3b, d, f).

The blockade of shikonin with zVAD showed no change in HA2X hyper-activation of PARP in all live cell phenotypes compared to drug treatment, with the exception of a reduction in the QN population (Fig. 4a, Fig. 2Sa–d). While most live cell phenotypes showed increased parthanatos (Fig. 4b, Figs. 1Sb–d, 2Sb–d), early apoptotic cells showed no change compared to drug alone or untreated cells (Fig. 4, Figs. 1Sa, i, 2Sa). In contrast DDR was only increased in the RIP1-dependent apoptotic cells compared to treated cells (Fig. 4c, Figs. 1Sb, j, 2Sb). All the dead phenotypes after zVAD blockade now showed reduced hyper-action of PARP compared to drug alone (Fig. 4a, Figs. 1Sm–p, 2Se–h). Parthanatos and DDR was unchanged after zVAD blockade in the all phenotypes (except DN) compared to drug or untreated cells respectively (Fig. 4b, c, Figs. 1Se–g, m–o, 2Se–g). However, the dead DN population showed increased parthanatos after zVAD blockade of shikonin compared to drug or untreated cells (Fig. 4b, Figs. 1Sh, p, 2Sh).

### Necrostatin-1 blockade of necroptosis

Necrostatin-1 is known to inhibit necroptosis [23, 27], interestingly more necrosis (15%) was detected along with early apoptotic cells compared to drug alone (Fig. 2d). The up-regulation of RIP3 (MFI 31,339, NS) was blocked by necrostatin-1 while the incidence of the necroptotic phenotype was increased (33% compared to 6% shikonin, Fig. 3c, g) [23, 27]. Live early apoptosis was also reduced (43%) while the level of RIP1-dependent apoptosis was maintained (13%, Fig. 3c, g). Such dead cells showed a reduction in late apoptosis in combination with a rise in the DN population (Fig. 3d, f, h).

Further analysis of necrostatin-1-shikonin treated live cells showed that hyper-activation of PARP was increased in the early apoptotic, RIP1-dependent apoptotic and DN populations compared to untreated cells (Fig. 4a, Figs. 1Si, j, l, 2Si, j, l). The blocked necroptotic phenotype and RIP1-dependent apoptotic cells had increased parthanatos but less DDR and via versa for the DN cells compared to untreated cells (Fig. 4b, c, Figs. 1Sa–d, 2Si–l). The incidence of QN was decreased in the RIP1-dependent apoptotic and DN populations compared to untreated cells (Figs. 2Sj, l, 1Sb, d). Analysis of dead cells showed that late apoptotic and blocked necroptotic phenotypes had increased hyper-activation of PARP compared to untreated cells (Fig. 4a, Figs. 2S, m, o, 1Se, g). The dead RIP1-dependent apoptotic cells showed no change in hyper-activation of PARP parthanatos and DDR compared to untreated controls (Fig. 4a, b, c, Figs. 2Sn, 1Sf). The late apoptotic and DN cells showed a decreased incidence of parthanatos with increased DDR compared to untreated cells (Fig. 4b, c, Fig. 2Sm, p).

### Blockade of apoptosis and necroptosis

Dual blockade of shikonin with zVAD and necrostatin-1 was investigated and the degree of early apoptosis was similar to that observed with zVAD blockade with a low level of cell death (Fig. 2e). Live and early apoptotic cells showed a high percentage of cells with RIP3 alone (33%) with reduced expression of RIP3 indicating that necroptosis was blocked (MFI 24,765, NS, Fig. 3i). Early apoptosis and RIP1-dependent apoptosis was low (11%, Fig. 3i). The incidence of the live DN population was enhanced as observed with zVAD and necrostatin-1 blockade (Fig. 3c, e, g, i). Such dead cells showed an increased presence of RIP1-dependent apoptosis and a similar level of late apoptosis compared to zVAD-shikonin treated cells (Fig. 3j).

The RIP1-dependent apoptotic and blocked necroptotic cells after dual blockade of shikonin both showed increased levels of hyper-activation of PARP and parthanatos with a reduction in DDR (Fig. 4a, b, Fig. 2Sr, s). While the early apoptotic and DN phenotypes now showed no change in the incidence of these populations compared to untreated cells (Fig. 4a, b, Fig. 2S, q, t). Such analysis of the late apoptotic population had more DDR but less parthanatos, while RIP1-dependent apoptotic cells showed no change compared to untreated cells (Fig. 4 b, c, Fig. 2Su, v). The blocked dead necroptotic phenotype had reduced levels of hyper-activation of PARP, parthanatos and DDR which was now increased (for hyper-activation of PARP) in the DN phenotype compared to untreated cells (Fig. 4a, b, c, Fig. 2Sw, x).

### Initiation of autophagy

**Fig. 5:**
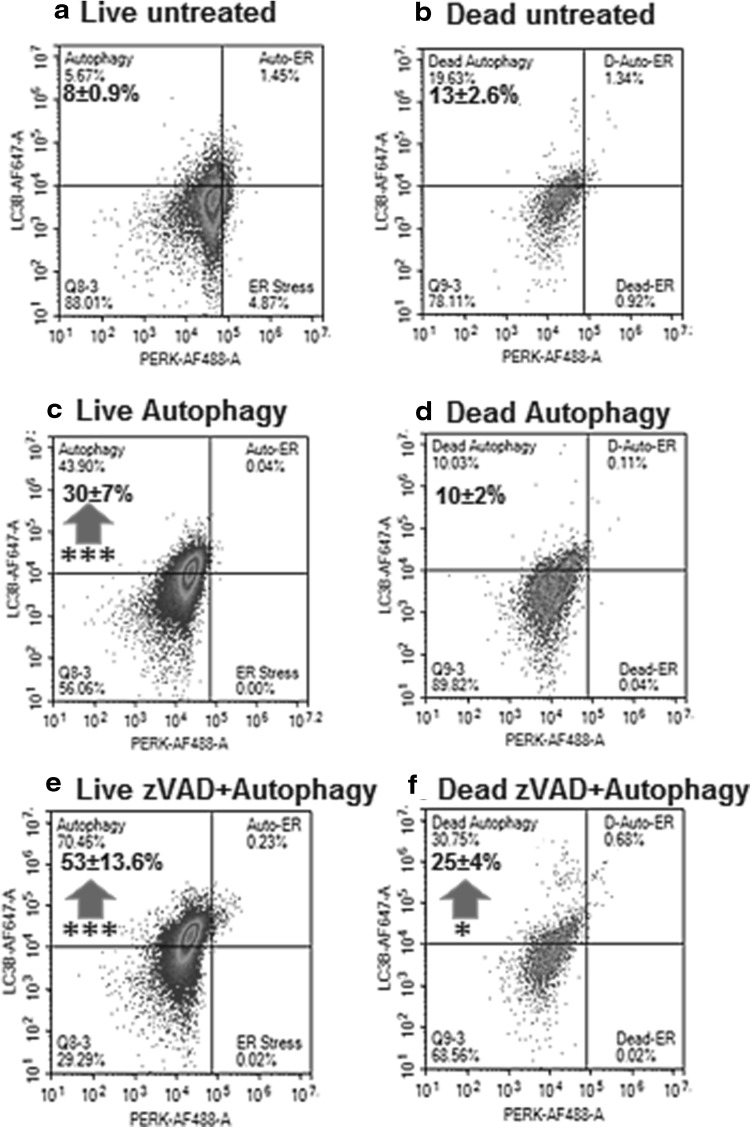
Autophagy assay. Untreated Jurkat cells or after CQ ± zVAD treatment for 24 h were analysed by gating on live and dead cells from a Zombie NIR vs. Caspase-3-BV650 dot-plot with live and dead of untreated cells (**a, b**), CQ treated (**c, d**) or CQ + zVAD treated Jurkat cells (**e, f**) were then analysed for autophagy (PERK^− ve^/LC3B^+ ve^) and ER stress (PERK^+ ve^/LC3B^− ve^) from a LC3B v PERK do-plot. n = 3, student *t* test NS (not significant), *P* < 0.05*, *P* < 0.01**, *P* < 0.001***, with arrows indicating change compared to untreated cells

**Fig. 6:**
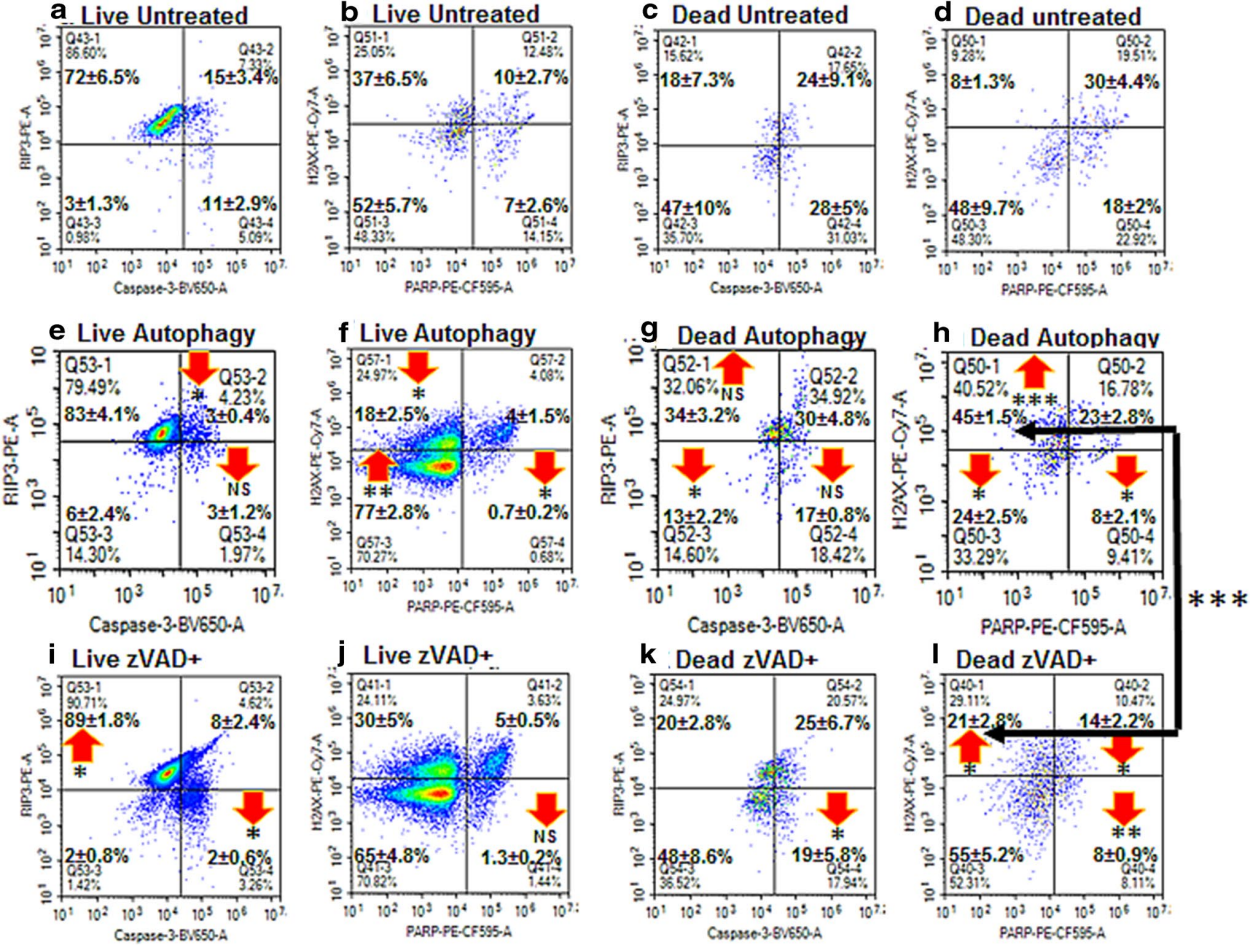
Apoptosis, hyper-activation of PARP, Parthanatos and DNA Damage assay on autophagic cells. Untreated Jurkat cells or after CQ ± zVAD treatment for 24 h were analysed by gating on live and dead cells from a Zombie NIR vs. Caspase-3-BV650 dot-plot, untreated cells or CQ ± zVAD treated autophagic live and dead LC3B^+ ve^/PERK^− ve^ were gated from a PERK-AF488 vs. LC3B-AF647 dot-plot. Autophagic cells were then gated on a RIP3-PE vs. Caspase-3-BV650 dot-plot to identify resting/necroptosis, RIP1-dependent apoptosis and apoptosis (live **a, e, i**, dead **c, g, k**) and a H2AX vs. PARP dot-plot to identify DDR, hyper-activation of PARP, parthanatos, and QN populations (live **b, f, i**, dead **d, h, l**). n = 3, student *t* test NS (not significant), *P* < 0.05*, *P* < 0.01**, *P* < 0.001***, with arrows indicating change compared to untreated cells, with black arrow indicating difference between dead autophagic ± zVAD

Treatment with a lysosome-autophagosome fusion inhibitor such as chloroquine (CQ) to initiate autophagy resulted in a small increase in late apoptosis compared to untreated cells (Fig. 2f). Increased LC3B expression above untreated cells was detected (30%) in live cells with no ER stress (PERK) and low levels of LC3B in dead cells (similar to untreated cells, Fig. 5a–d) [20, 21]. Gating on live LC3B^+ ve^/PERK^− ve^ cells showed lower levels of RIP1-dependent apoptosis and early apoptosis than untreated cells (Fig. 6a, e). Dead autophagic cells showed a twofold increase in the resting cell phenotype (RIP3^+ ve^/Caspase-3^− ve^, 34%, Fig. 6c, g). The incidence of late apoptosis and the DN populations was reduced (Fig. 6c, g).

Further analysis of H2AX and PARP after initiation of autophagy showed significantly less DDR and parthanatos in live autophagic cells, with the QN population significantly increased compared to untreated autophagic cells (Fig. 6b, f). Whilst dead CQ treated cells had significantly more DDR, less parthanatos and a lower incidence of the QN population than that observed in untreated autophagic cells (Fig. 6d, h).

### Blocking by zVAD

Blockade of apoptosis by zVAD during initiation of autophagy resulted in a small increase in necrosis (Fig. 2g). LC3B expression increased in live (53%) and dead after zVAD blockade (25%) cells with no ER stress compared to autophagic cells (Fig. 5e, f) [20, 29]. Gating on live LC3B^+ ve^/PERK^− ve^ cells showed the same low levels of RIP1-dependent apoptosis and early apoptosis observed with CQ (Fig. 6a, e, i). Dead autophagic cells treated with zVAD-CQ had a lower incidence of the resting or necroptotic phenotype (20%) compared to that observed in dead autophagic cells (Fig. 6c, g, k). Whilst the incidence of dead late apoptosis and RIP1-dependent apoptosis showed little change compared to autophagy alone despite blockade by zVAD (Fig. 6g, k). The incidence of the dead DN population was increased compared to that observed during autophagy (Fig. 6c, g, k).

Further analysis of live cell DDR and parthanatos showed a fall in parthanatos and increased DDR after initiation of autophagy with zVAD blockade compared to autophagic cells (Fig. 6b, f, j). Dead autophagic cells treated with zVAD-CQ had significantly less DDR (H2AX^+ ve^/PARP^− ve^) than autophagic cells (*P* < 0.001, as indicated by the black arrow) as well as a lower level of hyper-activated PARP (Fig. 6h, l).

## Discussion

The intracellular labelling of cells with fluorescently tagged antibodies to RCD defining target molecules, active caspase-3 (apoptosis), up-regulated RIP3 (necroptosis), up-regulated LC3B (autophagy), PARP (parthanatos), H2AX (DDR), PERK (ER Stress) in conjunction with a fixable live-dead cell stain gives the researcher detailed information about the distribution and incidence of multiple forms of RCD in live and dead cells simultaneously. This a major advance upon the information gained by other methodologies where there is no such discrimination [6–8, 16, 19–21, 23].

The use of a live-dead fixable stain with an active caspase-3 antibody allows the identification of early (Caspase-3^+ ve^/Viability^− ve^), late apoptosis (Caspase-3^+ ve^/Viability^+ ve^) and necrosis (Caspase-3^− ve^/Viability^+ ve^) [19, 23]. These populations were all observed after 24 h treatment with shikonin which is known to induce both apoptosis and necroptosis in Jurkat cells. So the addition of RIP3 permitted the identification of necroptosis by the up-regulation of RIP3 by shikonin above control levels in caspase-3 negative live cells. An increased incidence of live RIP1-dependent apoptosis was also detected and defined as positive for RIP3 and caspase-3 assuming that RIP1 which by definition is also present with RIP3 [6, 12–14, 19, 23]. Early and late apoptosis was also detected and defined not only by the presence of active caspase-3 but also the absence of RIP3 in the live and dead cells.

After zVAD blockade of shikonin induced apoptosis which resulted in an increase in necrotic cell death [19, 23], live cells showed an increased incidence of necroptosis coupled with an increase in the DN population compared to shikonin treatment. While dead cells showed a shift to the DN phenotype and away from that of apoptosis indicating that shikonin was still initiating apoptosis while zVAD was blocking the activation of caspase-3. Necrostatin-1 pre-treatment with shikonin resulted in no up-regulation of RIP3 indicating a blockade of necroptosis [19, 23, 27]. Blockade of necroptosis resulted in increased incidence of this population with a correspondingly lower level of early apoptosis than was expected. Shikonin with zVAD and necrostatin-1 blockade showed lower levels of early and late apoptosis with an increase in the incidence of blocked necroptotic cells which again displayed no up-regulation of RIP3. However, zVAD did not block dead cell RIP1-dependent apoptosis indicating that zVAD did not fully block the activation of caspase-3 via this signalling pathway in the presence of necrostatin-1.

Further discernible differences in the four live and dead RCD phenotypes were detected by the addition of PARP and H2AX antibodies allowing the researcher to distinguish a further 32 subpopulations of cells by measurement of DDR, parthanatos, hyper-activation of PARP and a QN population [7, 8]. Shikonin treatment increased parthanatos and H2AX hyper-activation of PARP in all live cell RCD phenotypes except early apoptotic cells. Dead cell RCD phenotypes also showed increased H2AX hyper-activation of PARP, while both apoptotic populations showed a decrease in parthanatos while other phenotypes showed an increase. Dead cell levels of DDR were increased by shikonin in late apoptotic cells but decreased in other phenotypes.

The zVAD blockade of shikonin resulted in no change in these levels of hyper-activation (except DN) and DDR (increased in RIP1-dependent apoptosis) in all live cell phenotypes, with mainly high levels of parthanatos observed in these populations. While the dead cell phenotypes after zVAD blockade of shikonin showed reduced levels of hyper-activation and parthanatos with no change in DDR levels, except increased DDR levels in both types of apoptosis. In contrast necrostatin-1 blockade of shikonin reduced levels of live cell hyper-activation of PARP in the live blocked necroptotic phenotype and increased levels in early apoptotic and DN cells compared to drug treatment. Parthanatos and DDR was reduced except were DDR was increased in live RIP1-dependent apoptosis and DN phenotypes compared to drug treatment. While hyper-activation of PARP was reduced in dead RIP1-dependent apoptosis and DN phenotypes there was no change observed in others after necrostatin-1 blockade of shikonin. In contrast dead cell parthanatos was unchanged while DDR levels increased in all dead cell phenotypes compared to drug treatment. Dual blockade of shikonin with zVAD and necrostatin-1 showed reductions of H2AX hyper-activation of PARP in all live and dead cell phenotypes and so almost cancelled out the effect of shikonin induced cell damage. This was also true of the levels of parthanatos observed in all dead cells and live early apoptosis and DN phenotypes, while an increase in parthanatos was observed in live RIP1-dependent apoptosis and the blocked necroptotic phenotype. While live cell DDR was reduced and unchanged in dead cell phenotypes. Thus it is possible by the use of this novel intracellular labelling of target antigen specific for each RCD of interest to show the mode of action of drugs and the mechanisms involved with blocking agents such as zVAD and necrostatin-1.

Autophagic treatment ± zVAD showed increased necrosis, late apoptosis and initiation of autophagy respectively [29]. Analysis of LC3B expressing live cells showed a decrease in both types of apoptosis, while after zVAD pre-treatment a decrease in early apoptotic cells was observed. Increased levels of DDR and parthanatos resulting in hyper-activation of PARP and thus NAD^+^ depletion with resultant autophagic and necrotic cell death have been previously reported [28]. Thus it is interesting to show in this study that live high LC3B expressing Jurkat cells displayed reduced levels of DDR and parthanatos indicating that the autophagic process enhanced cell health while zVAD reduced this affect [28]. However, dead autophagic cells showed an enhanced level of DDR with reduced levels of parthanatos while zVAD reduced the enhancement of DDR in dead cells. Thus the assay here showed a DDR related mechanism of the protective affect that autophagy has upon cells within live cells with resultant increase in dead cells.

This new approach for the measurement of cell death reveals the complexity of RCD by identifying 124 populations in live and dead cells and the complex shifts after blockade of shikonin by zVAD and necrostatin-1 in the incidence of defined RCD phenotypes of necroptosis, apoptosis and RIP1-dependent apoptosis in terms of DDR, parthanatos and hyper-activation of PARP revealing also the protective effects of autophagy on cell health via DNA repair. To our knowledge, this is the most comprehensive method for the investigation of cell death and as such it carries potential to expand detailed studies of cell death across both basic research and clinical settings.

## Electronic supplementary material

Below is the link to the electronic supplementary material.

**Supplementary Fig. 1S**. Hyper-activation of PARP, parthanatos and DNA Damage assay. After gating on live and dead cells from a Zombie NIR vs. Caspase-3-BV650 dot-plot untreated or shikonin treated live and dead Jurkat cells were analysed on a RIP3-PE vs. Caspase-3-BV650 dot-plot with the live early or dead late apoptosis phenotype (RIP3^-ve^/Caspase-3^+ve^) and analysed for (a, e, i, m), live or dead RIP1-dependent apoptosis (RIP3^+ve^/Caspase-3^+ve^, b, f, j, n), live or dead resting or necroptotic phenotype (RIP3^high+ve^/Caspase-3^-ve^, c, g, k, o), or live or dead double negative (RIP3^-ve^/Caspase-3^-ve^, d, h, l, p) respectively and such populations were then analysed for H2AX and PARP. n=3, student t test NS (not significant), *P* < 0.05*, *P* < 0.01**, *P* < 0.001***, with red arrows indicating change compared to untreated cells.

**Supplementary Fig. 2S**. Hyper-activation of PARP, parthanatos and DNA Damage assay. Jurkat cells were pre-treated zVAD (20 µM) and or Necrostatin-1 (60 µM) for 2 h then incubated with 0.5 µM shikonin for 24 h. After gating on live and dead cells from a Zombie NIR vs. Caspase-3-BV650 dot-plot treated live and dead Jurkat cells with zVAD and shikonin, necrostatin-1 with shikonin or zVAD with necrostatin-1 and shikonin were analysed on a RIP3-PE v Caspase-3-BV650 dot-plot. The live early or dead late apoptosis phenotype (RIP3^-ve^/Caspase-3^+ve^, a, e, i, m, q, u), live or dead RIP1-dependent apoptosis phenotype (RIP3^+ve^/Caspase-3^+ve^, b, f, j, n, r, v), live or dead blocked or necroptotic phenotype (RIP3^high+ve^/Caspase-3^-ve^, c, g, k, o, s, w), or live or dead double negative (RIP3^-ve^/Caspase-3^-ve^, d, h, l, p, t, x), respectively. Such live and dead populations were then analysed for H2AX and PARP. n=3, student t test NS (not significant), *P* < 0.05*, *P* < 0.01**, *P* < 0.001***, with red arrows indicating change compared to untreated cells.

## Abbreviations

CALR: Calreticulin
CQ: Choloroquine
DDR: DNA damage response
DN: Double negative
ER: Endoplasmic reticulum
HA2X: Histone A2 family X
Hyper-act PARP: Hyper-activation PARP
LC3B: Microtubule associated light chain B
MFI: Median fluorescence intensity
MPT: Mitochondrial permeability transition pore complex
Nec-1: Necrostatin-1
PARP: Poly (ADP-ribose) polymerase 1
PERK: protein kinase R-like endoplasmic reticulum kinase
QN: Quadruple negative
RCD: Regulated cell death
RIP1: Receptor-interacting serine/threonine-protein kinase 1
RIP1 APO: RIP1-dependent apoptosis
RIP3: Receptor-interacting serine/threonine-protein kinase 3
RT: Room temperature
zVAD: Carbobenzoxy-valyl-alanyl-aspartyl-[*O*-methyl]-fluoromethyl ketone
