# Supplementary figures and images for: Simultaneous polychromatic flow cytometric detection of multiple forms of regulated cell death
Source: Apoptosis. 2019 Feb 20;24(5):453–64. doi: 10.1007/s10495-019-01528-w (PMC6522464; doi:10.1007/s10495-019-01528-w)

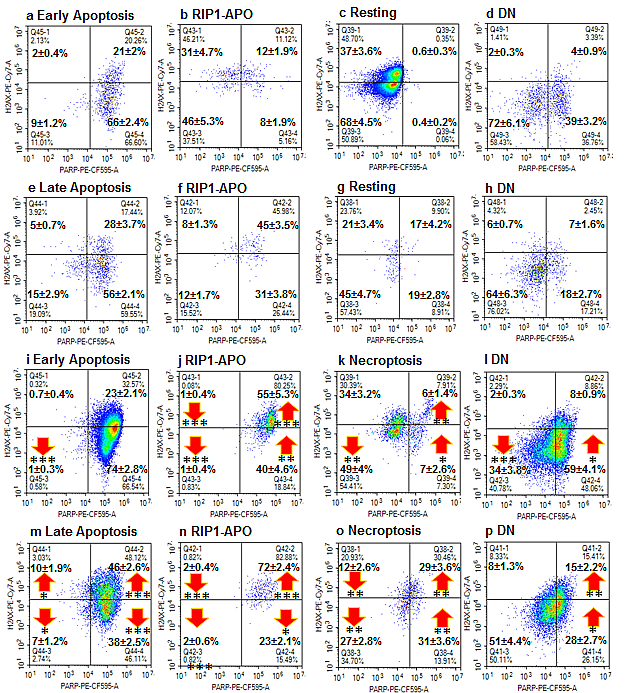

Supplement: Supplementary file 1 — Supplementary Fig. 1S. Hyper-activation of PARP, parthanatos and DNA Damage assay. After gating on live and dead cells from a Zombie NIR vs. Caspase-3-BV650 dot-plot untreated or shikonin treated live and dead Jurkat cells were analysed on a RIP3-PE vs. Caspase-3-BV650 dot-plot with the live early or dead late apoptosis phenotype (RIP3-ve/Caspase-3+ve) and analysed for (a, e, i, m), live or dead RIP1-dependent apoptosis (RIP3+ve/Caspase-3+ve, b, f, j, n), live or dead resting or necroptotic phenotype (RIP3high+ve/Caspase-3-ve, c, g, k, o), or live or dead double negative (RIP3-ve/Caspase-3-ve, d, h, l, p) respectively and such populations were then analysed for H2AX and PARP. n=3, student t test NS (not significant), P < 0.05*, P < 0.01**, P < 0.001***, with red arrows indicating change compared to untreated cells. [file 10495_2019_1528_MOESM1_ESM.tif]

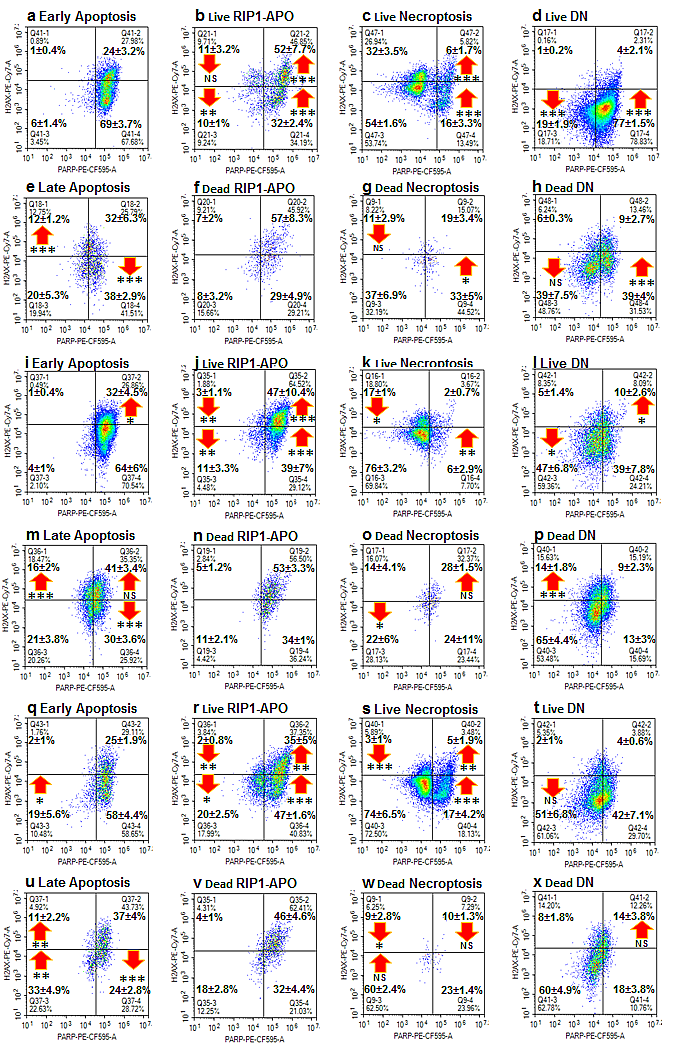

Supplement: Supplementary file 2 — Supplementary Fig. 2S. Hyper-activation of PARP, parthanatos and DNA Damage assay. Jurkat cells were pre-treated zVAD (20 µM) and or Necrostatin-1 (60 µM) for 2 h then incubated with 0.5 µM shikonin for 24 h. After gating on live and dead cells from a Zombie NIR vs. Caspase-3-BV650 dot-plot treated live and dead Jurkat cells with zVAD and shikonin, necrostatin-1 with shikonin or zVAD with necrostatin-1 and shikonin were analysed on a RIP3-PE v Caspase-3-BV650 dot-plot. The live early or dead late apoptosis phenotype (RIP3-ve/Caspase-3+ve, a, e, i, m, q, u), live or dead RIP1-dependent apoptosis phenotype (RIP3+ve/Caspase-3+ve, b, f, j, n, r, v), live or dead blocked or necroptotic phenotype (RIP3high+ve/Caspase-3-ve, c, g, k, o, s, w), or live or dead double negative (RIP3-ve/Caspase-3-ve, d, h, l, p, t, x), respectively. Such live and dead populations were then analysed for H2AX and PARP. n=3, student t test NS (not significant), P < 0.05*, P < 0.01**, P < 0.001***, with red arrows indicating change compared to untreated cells. [file 10495_2019_1528_MOESM2_ESM.tif]
